# Highly (H5N1) and Low (H7N2) Pathogenic Avian Influenza Virus Infection in Falcons Via Nasochoanal Route and Ingestion of Experimentally Infected Prey

**DOI:** 10.1371/journal.pone.0032107

**Published:** 2012-03-09

**Authors:** Kateri Bertran, Núria Busquets, Francesc Xavier Abad, Jorge García de la Fuente, David Solanes, Iván Cordón, Taiana Costa, Roser Dolz, Natàlia Majó

**Affiliations:** 1 Centre de Recerca en Sanitat Animal, Institut de Recerca i Tecnologia Agroalimentàries, Universitat Autònoma de Barcelona, Bellaterra, Barcelona, Spain; 2 Departament de Sanitat i Anatomia Animals, Universitat Autònoma de Barcelona, Bellaterra, Barcelona, Spain; 3 Roc Falcon S.L., Odèn, Lleida, Spain; Centers for Disease Control and Prevention, United States of America

## Abstract

An experimental infection with highly pathogenic avian influenza (HPAI) and low pathogenic avian influenza (LPAI) viruses was carried out on falcons in order to examine the effects of these viruses in terms of pathogenesis, viral distribution in tissues and viral shedding. The distribution pattern of influenza virus receptors was also assessed. Captive-reared gyr-saker (*Falco rusticolus* x *Falco cherrug*) hybrid falcons were challenged with a HPAI H5N1 virus (A/Great crested grebe/Basque Country/06.03249/2006) or a LPAI H7N2 virus (A/*Anas plathyrhynchos*/Spain/1877/2009), both via the nasochoanal route and by ingestion of previously infected specific pathogen free chicks. Infected falcons exhibited similar infection dynamics despite the different routes of exposure, demonstrating the effectiveness of in vivo feeding route. H5N1 infected falcons died, or were euthanized, between 5–7 days post-infection (dpi) after showing acute severe neurological signs. Presence of viral antigen in several tissues was confirmed by immunohistochemistry and real time RT-PCR (RRT-PCR), which were generally associated with significant microscopical lesions, mostly in the brain. Neither clinical signs, nor histopathological findings were observed in any of the H7N2 LPAI infected falcons, although all of them had seroconverted by 11 dpi. Avian receptors were strongly present in the upper respiratory tract of the falcons, in accordance with the consistent oral viral shedding detected by RRT-PCR in both H5N1 HPAI and H7N2 LPAI infected falcons. The present study demonstrates that gyr-saker hybrid falcons are highly susceptible to H5N1 HPAI virus infection, as previously observed, and that they may play a major role in the spreading of both HPAI and LPAI viruses. For the first time in raptors, natural infection by feeding on infected prey was successfully reproduced. The use of avian prey species in falconry husbandry and wildlife rehabilitation facilities could put valuable birds of prey and humans at risk and, therefore, this practice should be closely monitored.

## Introduction

Avian influenza (AI) is one of the most important biological threats, not only for poultry but also for other avian species and humans [1], [2]. Susceptibility to AI viruses varies greatly among wild bird and poultry species, as well as their possible role as vectors or reservoirs [3], [4]. Gallinaceous poultry are considered to be highly susceptible, whereas waterfowl have long been recognized as natural reservoirs, although they may show variable morbidity depending on the infective viral strain [5], [6].

Although AI typically courses as an asymptomatic infection in wild birds, recent highly pathogenic avian influenza (HPAI) epidemics resulted in unprecedented high mortality rates for certain wild bird species. In the past, HPAI viruses were rarely found in birds of prey and were restricted to only a few isolated cases [7], [8]. However, during recent H5N1 outbreaks, increasing number of birds of prey have been reported to be infected, probably as a result of improvements in sampling and diagnostic tools. It is worth highlighting that Hong Kong had a series of cases of natural infection of peregrine falcons (*Falco peregrinus*) with H5N1 in 2004, 2006 and 2008 [9]–[11], although other countries subsequently reported HPAI cases in different prey species, such as in Hodgson's hawk eagles (*Spizaetus nipalensis*) in Belgium [12], saker falcons (*Falco cherrug*) in Saudi Arabia [13] and, more recently in Saudi Arabia, houbara bustards (*Chlamydotis undulate macqueenii*),which interestingly infected falcons that came into contact with them [14].

Even though the number of AI natural cases in raptor species has gradually increased, data prevalence is still scarce. However, some countries have performed active AI surveillance of these species. In Sweden, neither HPAI nor low pathogenic avian influenza (LPAI) infections were found in white-tailed sea eagle (*Haliaeetus albicilla*) or peregrine falcons by standard screening using real time RT-PCR (RRT-PCR), and serology [15]. Besides this, 7.7% of the Falconiformes tested between August 2005 and February 2006 in the United Arab Emirates were seropositive [16]. It should be noted that in the Middle East, in addition to the circulation of the H5N1 viruses, there is also evidence of extensive circulation of LPAI viruses, mainly H9N2 viruses [17], [18]. The co-circulation of H9N2 and H5N1 subtypes of AI in these species may increase the risk of generating reassortant viruses with pandemic potential [19].

Falconry is an ancient tradition in the Arabian Peninsula that has spread worldwide, resulting in a strong trade of all species of falcons around the world. Nowadays, falconry is most popular in European countries such as The United Kingdom, Germany and Spain, in that order [20]. It is well known that migration of infected wild birds is one of the mechanisms in the spreading of AI viruses [21], thus many falcon species may contribute to the movement of both HPAI and LPAI viruses within, or between countries. Wild birds of prey are at an increased risk of acquiring AI viruses because they regularly feed on avian carcasses and diseased avian prey [22]–[24]. In falconry, birds of prey are kept in captivity and come into close contact with humans. Although there is still no direct evidence of virus transmission from falcons to humans, birds of prey could represent a bridging species for AI viruses and, consequently, the practice of falconry may pose an enhanced risk of transmission to humans and poultry. However, a recent study by Kohls *et al*. [25] indicates that the AI virus prevalence of prey birds from falconry is generally low, and that falconry birds which come into contact with AI viruses through their prey do not necessarily become infected, as in most cases the falconer does not allow them to eat the whole prey. However, concerning ornithophagous free ranging raptors, the risk of infection would be higher, since these usually feed on the whole prey. There is no evidence to confirm this so far.

To date, scarce experimental infections have been performed in birds of prey. Lierz *et al*. [26] studied the effects of H5N1 HPAI virus infection by performing an experimental vaccination trial on captive gyr-saker falcon hybrids (*F. rusticolis* × *F. cherrug*) via the oculo-oronasal route. Recently, an experimental infection in American kestrels (*Falco sparverius*) with various doses of H5N1 HPAI virus inoculated via the intranasal and intrachoanal route was performed [27]. Both studies showed that these birds are extremely susceptible to the H5N1 HPAI virus.

Although it is evident that birds of prey can be infected with HPAI viruses, their susceptibility to LPAI viruses still remains unclear, and the pathogenicity of both HPAI and LPAI viruses to these species has not been described extensively. Moreover, raptor's putative role as reservoirs in AI ecology and their potential to shed viruses need to be investigated. In the present study, the pathogenesis of HPAI and LPAI viruses in ¾ gyr-saker (*Falco rusticolus* × *Falco cherrug*) hybrid falcons was determined. The birds were experimentally inoculated via the nasochoanal route and by ingestion of virus-infected preys. Viral load distribution in several tissues and the extent and duration of viral shedding were also evaluated. In addition, localization of influenza virus receptors in different tissues was also assessed in order to identify the target cells of AI viruses in this species.

## Materials and Methods

### Ethics Statement

This study was carried out in strict accordance with the recommendations of the Ethical Commission of Animal Experimentation of the Autonomous Government of Catalonia (*Comissió d'Experimentació Animal de la Generalitat* (CEA), Permit Number: 5567). The protocol was approved by the Ethics Committee of Animal and Human Experimentation of the *Universitat Autònoma de Barcelona* (*Comissió d'Ètica en l'Experimentació Animal i Humana* (CEEAH), Permit Number: 1066). All manipulations were performed under sodium pentobarbital anesthesia, and every effort was made to minimize suffering. Welfare information and end point criteria are included in Text S1.

### Viruses

Two strains of AI virus were used: the HPAI isolate A/Great crested grebe/Basque Country/06.03249/2006 (H5N1) (H5N1 HPAI) and the LPAI isolate A/*Anas plathyrhynchos*/Spain/1877/2009 (H7N2) (H7N2 LPAI). The deduced amino acid sequence of the region coding for the cleavage site of the haemagglutinin molecule were PEIPKGSRVRR*GLF for the isolate H5N1 HPAI and PEIPKGR*GLF for the isolate H7N2 LPAI, being typical of HPAI and LPAI viruses, respectively. In addition, the H5N1 HPAI subtype demonstrated an intravenous pathogenicity index of 3.0 [28].

Stocks of the H5N1 HPAI and the H7N2 LPAI viruses were produced in 9-day-old embryonated specific pathogen free (SPF) chicken eggs. In both cases, the allantoic fluid was harvested at 48 hours post inoculation, aliquoted and stored at −80° C until use. Virus was diluted tenfold in phosphate buffer saline (PBS) for titration in 9-day-old embryonated SPF chicken eggs. The 50% egg lethal dose (ELD_50_) for H5N1 HPAI, and the 50% egg infective dose (EID_50_) for H7N2 LPAI, were determined using the Reed and Muench method [29].

### Animals

Juvenile (5–10 weeks old) male captive-reared ¾ gyr-saker (*Falco rusticolus* × *Falco cherrug*) hybrid falcons were obtained from a breeder (Roc Falcon S.L). From 3 to 10 weeks of age, falcons were imprinted by a person from the research group in order to minimize further stress. In order to assess that optimal health conditions existed, a complete blood cell count (CBC) was performed and a peripheral blood smear evaluated to rule out the presence of hemoparasites in all birds. Results of the CBC and blood smears were unremarkable. Also, an anti-parasite treatment (Baycox®) was carried out. In addition, non-diluted serum samples were collected to ensure that birds were serologically negative for AI antibodies by a competitive enzyme-linked immunosorbent assay (C-ELISA) (ID-VET, Montpellier, France). Furthermore, oropharyngeal and cloacal swabs were collected in order to make certain that falcons were negative for AI virus by real time RT-PCR (RRT-PCR). Birds were weighed and tagged with numbered aluminum leg bands, and brought to biosafety level 3 (BSL-3) in CReSA facility, where they were randomly distributed within the experimental groups and separately housed in negative-pressured isolators with HEPA-filtered air. It is worth highlighting that isolators were environmentally adapted for falcons' welfare (Text S1). Whole chick-preys, supplemented with vitamins were provided twice a day. Birds were adapted to their new environment for 5 days before experimental infection.

### Experimental design

The experimental design is summarized in Table 1. Seventeen falcons were distributed into five experimental groups (A to E). Groups A (n = 4) and B (n = 4) were challenged with 10^6^ EID_50_ of the H7N2 LPAI virus, whereas groups C (n = 3) and D (n = 4) were challenged with 10^6^ ELD_50_ of the H5N1 HPAI virus. Two falcons from group E were inoculated nasochoanally with PBS solution and served as negative controls.

**Table 1:** Experimental design of the study.

GROUPS	INOCULUM	TITER/N° CHICKS	INFECTION ROUTE	NUMBER OF ANIMALS
A	H7N2 LPAI	10^6^ EID_50_	Nasochoanal	4 (3+1)
B	H7N2 LPAI	2 chicks/falcon	Feeding	4
C	H5N1 HPAI	10^6^ ELD_50_	Nasochoanal	3
D	H5N1 HPAI	5 chicks/falcon	Feeding	4
E	PBS	–	Nasochoanal	2

LPAI, low pathogenic avian influenza; HPAI, highly pathogenic avian influenza; PBS, phosphate buffer saline; EID_50_, 50% egg infectious dose; ELD_50_, 50% egg lethal dose.

LPAI virus was A/*Anas plathyrhynchos*/Spain/1877/2009 (H7N2); HPAI virus was A/Great crested grebe/Basque Country/06.03249/2006 (H5N1).

Groups A and C were inoculated nasochoanally; in group A, one of the 4 falcons was not-inoculated and was referred to as the contact animal. Groups B and D were challenged via the natural feeding route with previously infected SPF chicks. Briefly, one-day-old SPF chicks, confirmed negative for AI virus by RRT-PCR, were inoculated via oculonasal route with either 10^6^ EID_50_ of the H7N2 LPAI virus or 10^6^ ELD_50_ of the H5N1 HPAI virus. At 3 days post-infection (dpi), chicks were confirmed positive for AI virus by performing a RRT-PCR on oropharyngeal and cloacal swabs. Two whole H7N2 LPAI-infected chicks and five whole H5N1 HPAI-infected chicks were used to infect each falcon from groups B and D, respectively. In order to estimate the viral load ingested by the falcons, viral titration in Madin-Darby Canine Kidney (MDCK) cells was performed on tissue homogenates from LPAI-infected and HPAI-infected chicks. Two out of three homogenates of trachea, lung, kidney and small intestine from H7N2 LPAI-infected chicks contained from 10^3.2^ to 10^7.2^ TCID_50_/g of tissue, whereas the viral load from homogenates of liver, lung, kidney and brain of all H5N1 HPAI-infected chicks reached titers from 10^6.5^ to 10^7.4^ TCID_50_/g of tissue. Inocula titers of all the experimental groups were verified by performing a RRT-PCR of both the original non-diluted viruses and the diluted inocula.

### Sampling

All falcons were monitored daily for the development of clinical signs, and oropharyngeal and cloacal swabs were obtained to measure viral shedding by RRT-PCR. Besides, blood samples were collected before euthanasia to detect AI antibodies by C-ELISA testing. As it was terminal, bleeding was done from the heart after previous anesthesia with intramuscular injection of ketamine/xylazine (10 g/kg body weight, Imalgene® 1000 and 1 g/kg body weight, Xilagesic® 2%). Mortality and mean death time (MDT) were calculated. Ethically euthanized and naturally dead falcons were necropsied to evaluate gross lesions and obtain samples for pathological and molecular studies. Negative control falcons and surviving infected falcons were euthanized at the end of the experiment (11 dpi for LPAI groups and 10 dpi for HPAI groups). Falcons were euthanized using intravenous sodium pentobarbital (100 mg/kg, Dolethal®,Vétoquinol, Cedex, France). Oropharyngeal and cloacal swabs, blood samples and tissue samples for molecular studies were stored at −80° C until further use.

### Histopathology

Necropsies and tissue sampling were performed according to a standard protocol. After fixation in 10% neutral buffered formalin and paraffin embedding, tissue sections were processed routinely for haematoxylin/eosin (H/E) staining. The following tissues were examined: esophagus, crop, proventriculus-ventriculus, duodenum, jejunum-ileum, cecum/cecal tonsil, rectum, pancreas, liver, kidney, adrenal gland, gonad, nasal turbinates, trachea, lung, heart, breast muscle, skin, bone marrow, spleen, bursa of Fabricius, thymus, brain, spinal cord and sciatic nerve.

### Virus detection by immunohistochemistry (IHC)

An immunohistochemical technique based on the Avidin-biotin complex immunoperoxidase system was performed as previously described [30], [31]. The primary antibody was a mouse-derived monoclonal commercial antibody against nucleoprotein (NP) of influenza A virus (IgG2a, Hb65, ATCC). As a secondary antibody, a biotinylated goat anti-mouse IgG antibody (GaMb, Dako E0433, Glostrup, Denmark) was used. Tissues previously demonstrated to be positive against nucleoprotein of influenza A virus by IHC were used as a positive control. Tissues from sham-inoculated animals were incubated without the primary antibody and served as a negative control. The following score was used in order to grade the staining in tissues: no positive cells (−), single positive cells (+), scattered groups of positive cells (++), widespread positivity (+++).

### Virus detection by RRT-PCR

Oropharyngeal and cloacal swabs were placed in 0.5 mL of Dulbecco's Modified Eagle's Medium (DMEM) with antibiotics. Additionally, tissue samples from trachea, lung, kidney and small intestine of H7N2 LPAI infected falcons, and from lung, kidney, duodenum/pancreas, liver and brain of H5N1 HPAI infected falcons were placed in 0.5 mL of PBS. Viral RNA was extracted with NucleoSpin® RNA Virus kit (Macherey-Nagel, Düren, Germany) following the manufacturer's instructions. A RRT-PCR assay was used to detect the viral *M* gene in Fast7500 equipment (Applied Biosystems, Foster City, CA, USA), using the primers and probe previously described [32], at a concentration of 400 nM for each primer and 300 nM for the TaqMan probe, the AgPath-ID one-step RT-PCR reagents (Applied Biosystems, Foster City, CA, USA) and 3 µL of eluted RNA in a total volume of 20 µL. The amplification conditions were as follows: reverse transcription at 48° C 10 min; initial denaturation at 95° C for 10 min and 40 PCR-cycles of 97° C for 2 sec and 61° C for 30 sec.

### Serology

A C-ELISA test was carried out in order to detect antibodies against the A nucleoprotein of AI virus using the commercially available kit ID Screen® Influenza A Antibody Competition (ID-VET, Montpellier, France), performed according to the manufacturer's instructions.

### Lectin histochemistry detection of influenza virus receptors

Lectin histochemistry was carried out in respiratory (nasal turbinates, trachea and lung) and digestive (proventriculus, duodenum, ileum, cecum and rectum) tracts of control falcons using the lectins *Maackia amurensis* agglutinin II (MAAII) and *Sambucus nigrans* agglutinin (SNA), which show affinity for α-2,3 (avian type) and α-2,6 (human type) receptors, respectively.

Lectin histochemistry was performed using previously described procedures [33] with minor modifications. Briefly, 3 µm-thick sections were dewaxed and treated with 3% H_2_O_2_ in methanol to eliminate endogenous peroxidase activity, washed with TNT (0.1 M Tris HCl, 0.15 M NaCl, pH 7.5) and blocked with TNB (TNT plus blocking reagent) (Perkin Elmer, US) for 30 minutes at room temperature (RT). Tissue sections were then incubated with biotynilated SNA (10 µg/ml) and MAAII (15 µg/ml) (Vector Laboratories Inc, CA, US) in TNB at 4° C, overnight. After washing with TNT, sections were incubated with streptavidin-horse radish peroxidase (SA-HRP) 1∶100 for 1 hour, and again incubated with SA-HRP for 30 min at RT. The reaction was developed with diaminobenzidine (Sigma-Aldrich, MO, US) at RT for 30 seconds followed by counterstaining with Mayer's haematoxylin. To rule out the non-specific binding of lectins, two sequential slides were used as negative controls. One slide was pretreated with neuraminidase, which cleaves both α-2,3 and α-2,6 residues, as previously described [33], and the other was incubated with PBS instead of the lectins. Negative controls consisted of the substitution of the lectin with a TNB buffer. Human, pig and mice tissue samples were used as positive controls because of previous publications reporting the lectin pattern of staining of these species [33]–[36]. For each slide, and in order to compare receptor expression patterns among the tissues included in this study, the relative intensity of receptor expression was scored based on the percentage of cells in a section showing positivity, and was graded as: no positive cells (−), single positive cells (+), scattered groups of positive cells (++), widespread positivity (+++).

### Statistical analysis

Oral viral shedding obtained from RRT-PCR was analyzed by Kruskal-Wallis test for significant differences (*p*<0.05) between H7N2 and H5N1 groups and between routes of infection. The statistical tests were performed using the Statistical Package for the Social Sciences for Windows Version 17.0.

## Results

### Clinical signs and Mortality

There were no relevant differences between nasochoanally inoculated animals and animals challenged via the natural feeding route in both H5N1 HPAI and H7N2 LPAI groups. Falcons from both H5N1 HPAI challenged groups (groups C and D) showed first clinical signs at 5 dpi which consisted of depression, apathy, impaired respiration and, above all, slight neurological signs that in a matter of hours turned into moderate or severe, and included torticollis, head tilt, ataxia, circling, incoordination, leg/wing paralysis, opisthotonus and tremors (Movie S1). Some birds were also found recumbent and unresponsive. Following the endpoint criteria established in the ethical protocol, one falcon per group was ethically euthanized at 5 dpi. At 6 dpi, one falcon infected by feeding and showing severe neurological signs was also ethically euthanized, while at 7 dpi one falcon per group was found dead. Remaining falcons (one per group) survived until the end of the experiment at 10 dpi and did not develop clinical signs. To sum up mortality data, two out of three nasochoanally inoculated falcons, and three out of four falcons challenged by feeding died between 5 and 7 dpi, and the MDT was 6 dpi. Neither morbidity nor mortality was observed in the negative control group (group E) or in the H7N2 LPAI groups (groups A and B).

### Gross findings

**Figure 1:**
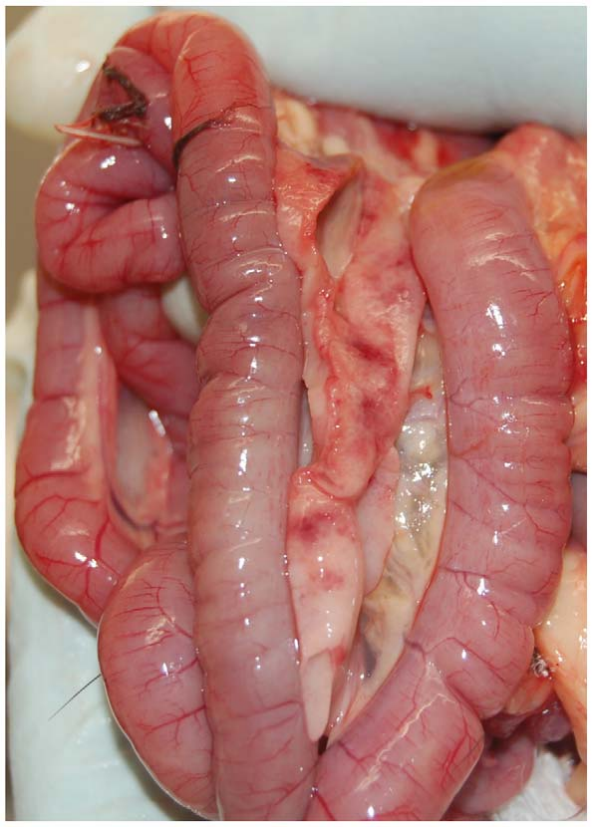
Pancreatic macroscopic lesions from a falcon experimentally infected with highly pathogenic avian influenza virus H5N1. Multifocal hemorrhagic necrosis in the pancreas of a falcon infected via the nasochoanal route with A/Great crested grebe/Basque Country/06.03249/2006 H5N1 HPAI virus (5 dpi).

Findings associated with AI virus infection were only observed in some of the H5N1 HPAI infected falcons (groups C and D). Lesions were very similar regardless of the infection group and day of necropsy, being pancreas, proventriculus, ventriculus and brain the most affected organs. Regarding the nasochoanal group (group C), multifocal hemorrhagic necrosis in the pancreas was found in the falcon necropsied at 5 dpi (Figure 1), while the falcon found dead at 7 dpi showed multifocal petechia on the proventriculus-ventriculus junction mucosa, as well as brain and eyelid congestion. The surviving falcon from this group also showed multifocal petechia in the proventriculus-ventriculus junction mucosa at time of necropsy (10 dpi). Concerning the prey ingestion group (group D), the falcon necropsied at 5 dpi did not show significant lesions, whereas multifocal petechia on the proventriculus-ventriculus junction mucosa and brain and eyelid congestion were found in the falcon euthanized at 6 dpi. In this group, the falcon found dead at 7 dpi also showed multifocal petechia on the proventriculus-ventriculus junction mucosa and multifocal hemorrhagic necrosis in the pancreas. No gross lesions were observed in the H5N1 HPAI infected falcon of the prey ingestion group euthanized at 10 dpi.

### Histopathological findings

Histological lesions and influenza A viral antigen (NP) were only observed in H5N1 HPAI challenged falcons (groups C and D) (Tables 2 and 3). Neither significant lesions nor positive IHC staining were observed in H7N2 LPAI challenged animals (groups A and B), or in the control birds. Tissues not present in Tables 2 or 3 appeared overtly normal on histopathological analysis and did not show positive IHC staining.

**Table 2:** Average distribution of nucleoprotein antigen, as determined by immunohistochemistry, in tissues sampled from falcons infected via the feeding route with A/Great crested grebe/Basque Country/06.03249/2006 (H5N1) HPAI virus.

TISSUE	2VN6	4AN6	3ES4	6TN14	PREDOMINANT CELL TYPES	ASSOCIATED LESION
	5 dpi	6 dpi	7 dpi	10 dpi		
Pancreas	−	−	−	−	−	NSL
Kidney	+	−	−	−	Collecting tubular epithelial cells	NSL
Nasal turbinates	nd	+	+	−	Lateral nasal gland epithelial cells	NSL
Trachea	−	+	−	−	Pseudostratified epithelial cells	Necrosis of pseudostratified epithelium. Mild focal tracheitis.
Lung	+++	−	nd	nd	Bronchial epithelial cells, goblet cells	NSL
Brain	+++	+++	++	+	Neurons, ependymal cells, glial cells, endothelial cells	Malacia in cortex. Necrosis of ependymal cells of ventricles and epithelial cells of choroid plexus. Perivascular cuffing. Endothelia hypertrophia. Chromatolysis of Purkinje cells.
Spinal cord	−	−	−	−	−	NSL

− = no positive cells; + = single positive cells; ++ = scattered groups of positive cells; +++ = widespread positivity.

NSL, no significant lesions; nd, not determined.

**Table 3:** Average distribution of nucleoprotein antigen, as determined by immunohistochemistry, in tissues sampled from falcons infected via the nasochoanal route with A/Great crested grebe/Basque Country/06.03249/2006 (H5N1) HPAI virus.

TISSUE	1VN6	4CS1	1US18	PREDOMINANT CELL TYPES	ASSOCIATED LESION
	5 dpi	7 dpi	10 dpi		
Pancreas	+	+	−	Acinar cells	Lytic necrosis. Heterophilic infiltrate.
Kidney	+	−	−	Collecting tubular epithelial cells	NSL
Nasal turbinates	−	nd	−	−	NSL
Trachea	−	−	−	−	NSL
Lung	+	+	+	Bronchial epithelial cells, goblet cells	NSL
Brain	+++	+++	−	Neurons, ependymal cells, glial cells, endothelial cells	Malacia in cortex. Necrosis of ependymal cells of ventricles and epithelial cells of choroid plexus. Perivascular cuffing. Chromatolysis of Purkinje cells. Endothelial hypertrophy.
Spinal cord	++	−	−	Neurons, ependymal cells, glial cells	Malacia in grey matter. Necrosis of the ependyma and neuropil.

− = no positive cells; + = single positive cells; ++ = scattered groups of positive cells; +++ = widespread positivity.

NSL, no significant lesions; nd, not determined.

**Figure 2:**
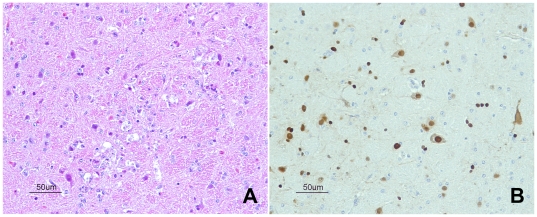
Brain tissue from an experimentally H5N1 HPAI virus-inoculated falcon dead at 5 dpi. **A.** Focal area of malacia in the cortex (HE stain). **B.** Immunohistochemical staining of influenza A virus antigen in brain tissue.

The most severely affected organ was the brain (Tables 2 and 3). The main findings consisted in moderate (5–6 dpi) to severe (7 dpi) multifocal areas of malacia in the cortex, present in all hemispheres of the brain, associated with spongiosis of the neuropil, chromatolysis, gliosis and caryolysis (Figure 2A). Vascular endothelial swelling was also observed, especially at 5–6 dpi. Evident severe necrosis of ependymal cells of the ventricles and epithelial cells of the choroid plexus was present in almost all falcons. The cerebellum frequently showed multifocal areas of moderate to severe chromatolysis of Purkinje neurons, sometimes associated with necrosis of the Purkinje cell layer and non-suppurative inflammatory infiltrate. Severe non-suppurative choroiditis was observed in two falcons of the prey ingestion group, particularly in the falcon found dead at 7 dpi and in the falcon euthanized at 10 dpi, as well as in the falcon found dead at 5 dpi of the nasochoanal group. Moderate multifocal areas of perivascular cuffing were present, being severe in the falcon from the prey ingestion group necropsied at 10 dpi. This falcon had the same histopathological findings as the neurologically affected falcons, whereas the falcon from the nasochoanal group necropsied at 10 dpi did not show any significant lesions in the brain. Significant microscopic lesions were seen in the spinal cord of the falcon from the nasochoanal group necropsied at 5 dpi, where severe malacia in the grey matter, and severe gliosis and necrosis of the ependym and neuropil surrounding the medullary canal were observed. Interestingly, almost all lung tissues showed positive IHC staining in bronchi, in particular bronchial epithelial cells and goblet cells. In general, antigenic staining was mainly nuclear and also often cytoplasmic in distribution and correlated well with histopathological findings (Figure 2B).

### Avian influenza virus detection by RRT-PCR

**Figure 3:**
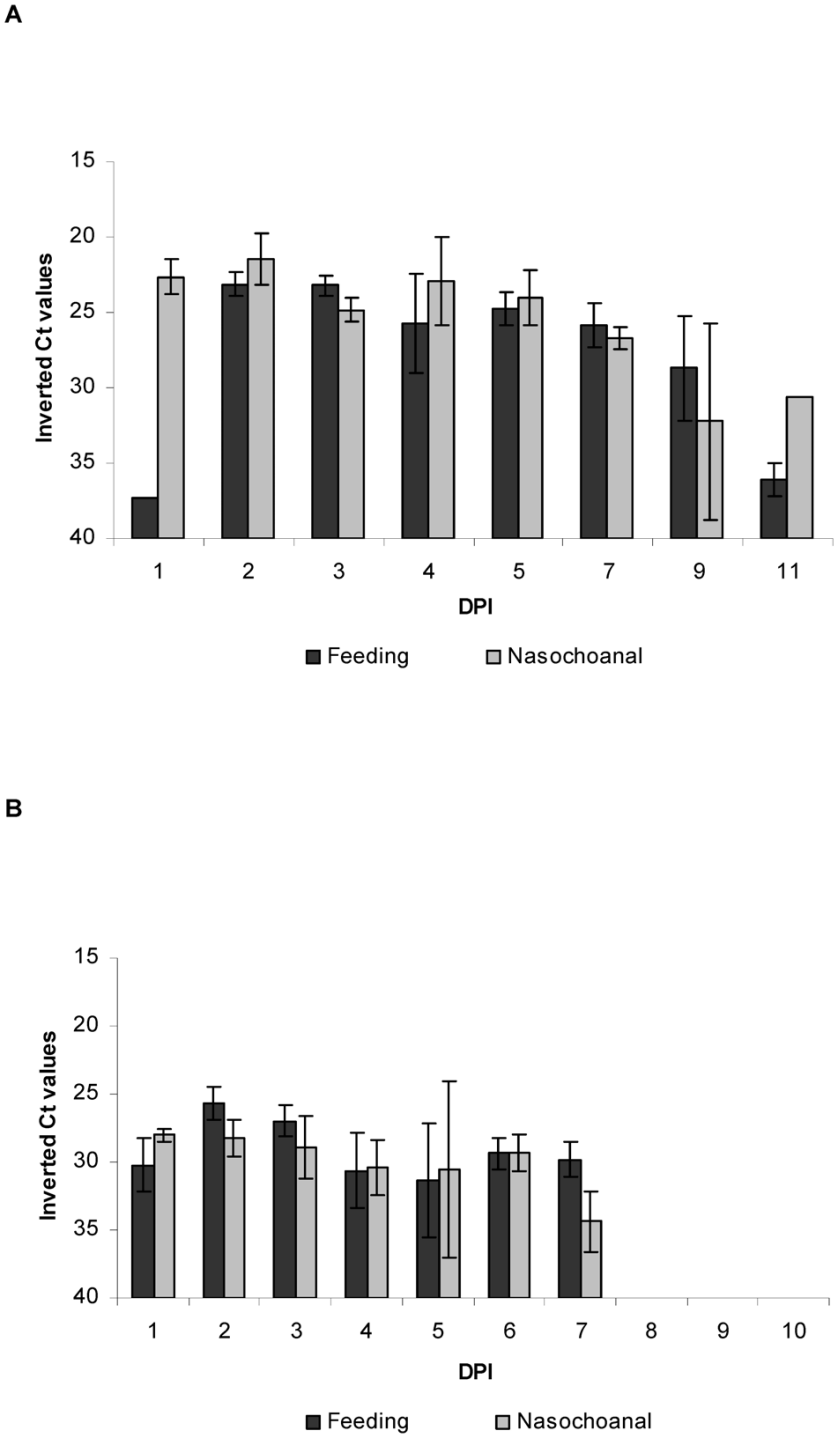
Oral shedding from experimentally infected falcons with avian influenza virus. Viral RNA shedding detected by RRT-PCR in oropharyngeal swabs of falcons infected via the feeding route or via the nasochoanal route. Ct, cycle of threshold. **A.** Falcons infected with A/*Anas plathyrhynchos*/Spain/1877/2009 (H7N2) LPAI virus and euthanized at 11 dpi. **B.** Falcons infected with A/Great crested grebe/Basque Country/06.03249/2006 (H5N1) HPAI virus.

Real time RT-PCR was performed on oropharyngeal and cloacal swabs, and on various tissues obtained at necropsy. In both H7N2 LPAI-infected groups (groups A and B) (Figure 3A), viral RNA was only detected on oropharyngeal swabs. Regarding H7N2 LPAI prey-infected falcons (group B) the amount of viral RNA detected orally was consistent and reached minimum cycle of threshold (Ct) values of 22.29 at 2 dpi. Besides, viral RNA was already detected at 1 dpi in one animal, with a Ct value of 37.31. H7N2 LPAI viral RNA was detected in all falcons until the end of the experiment (11 dpi), although amounts of viral RNA were evidently declining. Regarding falcons inoculated via the nasochoanal route (group A), detection was similar to that observed in the feeding group, although quantities of viral RNA declined more rapidly. No viral RNA was detected in swabs from the contact animal during the whole experiment.

For H5N1 HPAI infected falcons (groups C and D) (Figure 3B), viral RNA detection had a different profile than for that of H7N2 LPAI infected animals. Regarding oropharyngeal swabs, both nasochoanally and feeding infected falcons showed viral RNA from 1 dpi until prior to death, peaking at 2 dpi (Ct 24.08). Falcons that survived until the end of the experiment (10 dpi) stopped shedding at 7 dpi. In contrast with H7N2 LPAI falcons, H5N1 HPAI infected animals showed some viral RNA detection in cloacal swabs. In particular, the falcon from the feeding group that was euthanized at 5 dpi showed low amounts of virus (Ct 33.15) at the time of necropsy, as well as two falcons from the nasochoanal group: the one that was euthanized at 5 dpi showed virus from 3 to 5 dpi (Ct 33.77, Ct 37.96 and Ct 29.21), and the one found dead at 7 dpi had a Ct value of 31.51 at 5 dpi.

Results of viral detection by RRT-PCR from the tissues are shown in Table 4. In general, all falcons that showed clinical signs and died, or were ethically euthanized had positive results for almost all the selected tissues. The highest amounts of viral RNA were detected in the brain. The surviving falcon's brain from the feeding group was positive for RRT-PCR.

**Table 4:** Viral RNA in tissues of falcons infected with A/Great crested grebe/Basque Country/06.03249/2006 (H5N1) HPAI virus.

INFECTION ROUTE, ANIMAL ID	DAY OF DEATH	RNA in tissue, Ct value				
		Lung	Kidney-adrenal gland	Duodenum-pancreas	CNS	Liver
**Feeding**						
2VN6	5	24.23	25.42	25.58	15.82	undet
4AN6	6	28.77	undet	34.92	14.45	undet
3ES4	7	29.23	32.67	36.31	14.73	undet
6TN14	10	undet	undet	undet	19.22	undet
**Nasochoanal**						
1VN6	5	23.90	24.56	26.16	14.43	29,36
4CS1	7	26.48	29.38	28.45	17.84	undet
1US18	10	undet	undet	undet	undet	undet

Ct, cycle of threshold; CNS, central nervous system; undet, not detected by RRT-PCR.

The statistical analysis performed on the results of the oropharyngeal swabs until 7 dpi (both individual and mean Ct) showed no significant differences between routes of infection for both H5N1 HPAI and H7N2 LPAI groups (*p*>0.05), whereas amounts of viral RNA of H7N2 LPAI groups were significantly higher than in H5N1 HPAI groups (*p*<0.05).

### Serology

With the exception of the contact bird, all H7N2 LPAI infected falcons were seropositive at the end of the experiment (11 dpi). Concerning H5N1 HPAI infected falcons, only serum samples from 10 dpi were seropositive. No seroconversion was observed in the negative control falcons (group E).

### Lectin histochemistry detection of influenza virus receptors

**Figure 4:**
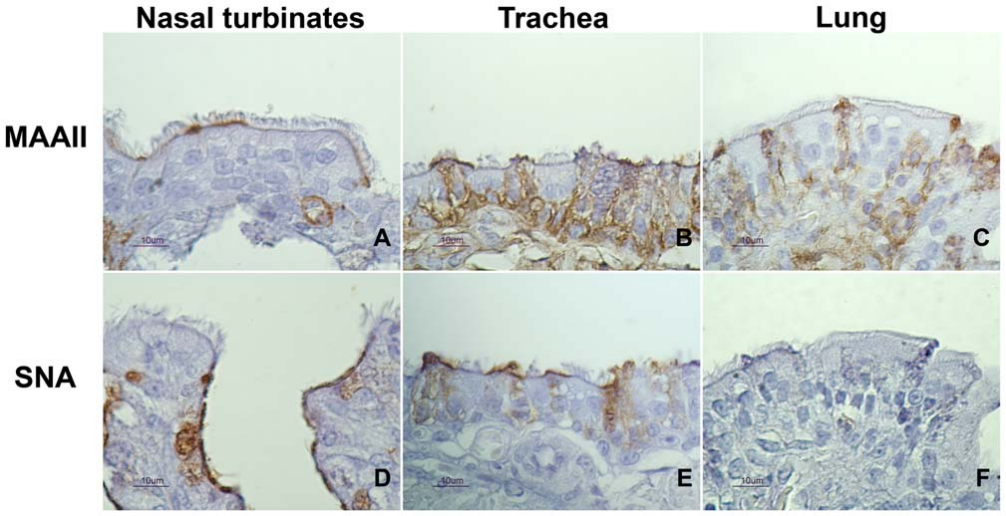
Distribution of α-2,3 and α-2,6 receptors in the respiratory tract of falcons demonstrated by means of MAAII and SNA lectin histochemistry. **A.** Nasal turbinates stained by MAAII lectin. **B.** Trachea stained by MAAII lectin. **C.** Lung stained by MAAII lectin. **D.** Nasal turbinates stained by SNA lectin. **E.** Trachea stained by SNA lectin. **F.** Lung stained by SNA lectin.

**Figure 5:**
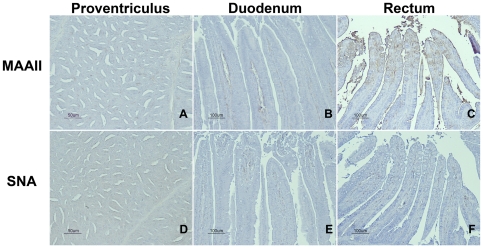
Distribution of α-2,3 and α-2,6 receptors in the digestive tract of falcons demonstrated by means of MAAII and SNA lectin histochemistry. **A.** Proventriculus stained by MAAII lectin. **B.** Duodenum stained by MAAII lectin. **C.** Rectum stained by MAAII lectin. **D.** Proventriculus stained by SNA lectin. **E.** Duodenum stained by SNA lectin. **F.** Rectum stained by SNA lectin.

Lectin immunohistochemistry was carried out in the respiratory and digestive tracts of control falcons in order to assess the distribution pattern of SNA (α-2,6) and MAAII (α-2,3). Regarding respiratory tract (Figure 4), moderate expression of α-2,6 was observed in ciliated epithelial cells and mucous gland cells of the respiratory tract of nasal turbinates and in salivary gland epithelium. Mild expression of α-2,6 receptors was observed in ciliated epithelial cells, mucous gland epithelium and goblet cells of the trachea. However, expression of receptors was predominantly α-2,3 (avian type) on the respiratory tract, being as follows: strong in bronchial epithelial cells; moderate in ciliated epithelial cells and goblet cells of the trachea; and mild in ciliated epithelial cells of the respiratory tract of nasal turbinates and in nasal gland epithelium, and in mucous gland epithelium of the trachea. Regarding digestive tract (Figure 5), strong α-2,3 expression was noted in enterocytes of the rectum, and mild α-2,3 expression was observed in goblet cells of the same region. Other cell types, such as endothelial cells, macrophages and lymphocytes, gave mild positive results for both α-2,3 and α-2,6 receptors in the cecum/cecal tonsil and rectum.

## Discussion

This is the first experiment which demonstrates that falcons can be infected with both HPAI and LPAI viruses, not only via the nasochoanal route but also by feeding on infected prey. For both viruses, infected falcons exhibited similar infection dynamics despite the different routes of exposure, demonstrating that ingestion of infected-SPF chicks is as effective as direct nasochoanal route to produce infection. To the best of our knowledge, this is the first study demonstrating that the consumption of infected prey is a viable route of transmission for both HPAI and LPAI viruses in falcons. Other studies have addressed the role of feeding on influenza-infected prey in other animal species [23], [24], [37]. Infectious dose to which the falcons were experimentally exposed via feeding on infected-SPF chicks could be comparable to the infectious dose to which wild falcons would be exposed during feeding on infected wild preys. This situation could also be feasible in falcons raised for falconry or in wild falcons clinically-admitted in wildlife rehabilitation centers when fed on AI infected preys (either live-bird market or wild birds). Therefore, this practice should be closely monitored.

The high pathogenicity of the H5N1 HPAI strain used in the present study is in agreement with that obtained in other H5N1 HPAI experimental infections in falcons [26], [27]. In our study, 5 out of 7 falcons died between 5 and 7 dpi; whether the two surviving falcons would have died in a longer experiment (at some point after the 10 dpi) remains unclear. Nevertheless, clinical signs of H5N1 HPAI infected animals were very acute and extremely severe, similar to those observed in Hall's study [27]. Such neurologic disorders would be feasible in natural infected falcons; these signs could be seen in free range birds under surveillance, and would certainly be noticed in animals under captivity. However, evident external lesions that are expected in AI-infected gallinaceous species, such as cutaneous hemorrhages on the legs [3], were not observed in falcons and, thus, may be overlooked during routine necropsy when AI is not suspected. Viral H5N1 HPAI RNA in tissues correlated well with IHC results, the brain being the most affected organ. Indeed, falcons only demonstrated severe neurological signs prior to death and only after necropsy and histopathological studies did some of the other tissues exhibit significant lesions. The feeding H5N1 HPAI infected falcon that survived until the end of the experiment could possibly have demonstrated clinical signs later on, since the virus was detected in the CNS by both IHC and RRT-PCR, and in the lungs by IHC.

Viral shedding was considered mainly oral for H5N1 HPAI infected falcons, being consistent and lasting for one week. In both experimental studies with H5N1 HPAI in falcons performed up to date [26], [27] oral shedding seemed to be predominant over cloacal shedding. In addition, the only viral shedding route observed in H7N2 LPAI infected falcons was the oral one, which was significantly higher and lasted longer (up to 11 dpi) than in H5N1 HPAI infected animals. Moreover, the distribution pattern of influenza virus receptors seems to be in agreement with the pattern of viral shedding observed. The presence of avian-type receptors (α-2,3) in the nasal turbinates, trachea and bronchial epithelium, and their absence in other parts of the lung, support previous findings regarding domestic birds that AI viruses mainly localize in the upper respiratory tract [5], [38], and thus, successful oral shedding is detected. The scarce cloacal shedding observed in some H5N1 HPAI infected falcons is in accordance with the absence of avian-type receptors in the intestinal tract, which was only expressed in rectum.

Given that falcons can shed a considerable amount of AI virus before the appearance of overt clinical signs or death (if so), this species may contribute to viral transmission within the geographical limits in free-living birds, or to a local outbreak when reared in outdoor operations. Therefore, infected falcons shedding AI virus could represent a risk for humans and other valuable bird species when admitted in wildlife rehabilitation centers or during shipping for falconry trade.

Raptors are at the top of their food chain, representing a natural surveillance system that target those subjects more likely to have had HPAI virus exposure. However, the possible introduction of HPAI or LPAI viruses in raptor populations could have a negative impact on already threatened species. Therefore, surveillance could be an invaluable tool in studying the epidemiological situation of AI viruses in raptor and other related wild bird populations. Data obtained in the present study indicates that oropharyngeal swabs can be successfully used for virus detection in falcon surveillance programs, as is also recommended for other species [21], [39]. In addition, not only brain, but also pancreas specimens are useful for AI virus detection and histopathological diagnosis.

In conclusion, our observations suggest that gyr-saker hybrid falcons are highly susceptible to infection with the H5N1 HPAI virus used in this study, and that they may play a major role in spreading AI viruses, given that a prolonged and consistent viral shedding has been demonstrated, especially with the H7N2 LPAI virus used in this study. Therefore, this species, whether wild or in captivity, should be included in passive surveillance programs, in order to prevent risk to humans and other wild bird species, and to minimize the threat of spreading, particularly of HPAI viruses within and among countries via animal trade or natural movements.

## Supporting Information

Text S1 **Welfare information and end point criteria established for the experimental infection.**
(DOC)

Movie S1 **Clinical signs in a falcon experimentally infected via the feeding route with highly pathogenic avian influenza virus H5N1.** Severe neurological signs like torticollis, ataxia, circling, incoordination and opisthotonus.
(AVI)
