# Supplementary material for: Highly (H5N1) and Low (H7N2) Pathogenic Avian Influenza Virus Infection in Falcons Via Nasochoanal Route and Ingestion of Experimentally Infected Prey
Source: PLoS One. 2012 Mar 9;7(3):e32107. doi: 10.1371/journal.pone.0032107 (PMC3302889; doi:10.1371/journal.pone.0032107)
Supplement: Text S1 — Welfare information and end point criteria established for the experimental infection. (DOC) [file pone.0032107.s001.doc]

After hatching, an average of twenty SPF chicks per each virus-group was randomly allocated in a negative-pressurized isolator. At 1 day of age, oculo-nasal LPAI and HPAI virus-inoculation were performed. After inoculation, SPF chicks were daily monitored for the development of clinical signs. Following the CEEAH and CEA recommendations, each clinical sing observed was scored from 0-3, as shown in Table 1. In order to avoid unnecessary suffering and/or pain of the animals used in the experiment, end point criteria were established, as shown in Table 2. The decision of euthanizing or not the bird was taken based on the scoring obtained from Table 1. For instance, following Table 1 scoring, a bird that was showing slight apathy (score 2), stained cloacal feathers (score 2) and evident gasping (score 2) was scored as 6 on Table 2, and therefore possible euthanasia was considered. A bird that, following Table 1 scoring, showed stained cloacal feathers (score 2), prostration (score 3) and pedaling (score 3) was scored as 10 on Table 2, because when a bird had more than one score 3 on Table 1, all the scores 3 changed to score 4 on Table 2; in this case the decision would be to euthanize the bird. In addition, all animals that showed a comatose status were euthanized.

Handling of the animals was always performed by qualified personnel accredited by the Federation of European Laboratory Animal Science Associations. All procedures were carried out restraining the birds correctly, safely and rapidly in order to avoid causing injuries and to minimize stress to the birds.

Regarding LPAI-infected-chicks, no clinical signs were observed and, at 3 days post-infection (dpi), they were euthanized by cervical dislocation. Regarding HPAI-infected-chicks, at 1 dpi no clinical signs were observed, whereas at 2 dpi all birds except one were found dead; the surviving chick was showing severe depression and was euthanized by cervical dislocation. When used for falcon’s feeding, naturally dead or euthanized chickens were half-opened and provided to the falcons.

The monitoring of clinical signs and the end point criteria established for the falcons were based on Tables 1 and 2, as explained above for the chicks. In the same way, handling of the animals was always performed by qualified personnel accredited by the Federation of European Laboratory Animal Science Associations. All procedures were carried out restraining the birds correctly, safely and rapidly in order to avoid causing injuries and to minimize stress to the birds.

Further details regarding the isolators and the environmental enrichment are provided. The negative-pressurized isolators used in the study are 1500x800x850 mm (wxdxh), with a 1.2 m^2^ surface floor and 1 m^3^ volume. They are provided with a deep tank (100 L) of a sodium hypochlorite solution that isolates the outside from the inside, and that, at the same time, allows the exchange of materials between the outside and the inside of the isolator. Besides, HEPA-filtered air systems guarantee negative pressure conditions. In addition, temperature and light control in accordance with the age and the species studied are provided. In our case, 1-day-old chicks were at 29º C, whereas falcons were at 19º C. Regarding light/dark cycle, both chicks and falcon’s isolators were programmed for 12 hours light-dark. Relative humidity was set up in 40-60 %.

In the chick’s isolators, a layer of cardboard was used as bedding material to cover the surface of the isolator’s floor, in order to avoid eventual hurting of the birds. The chicks were fed *ad libitum* with commercial compound feed suitable for their age in stainless steel feeders, and running water in bell drinkers and nipples suitable for day-old chicks.

As already indicated in the manuscript, it is worth highlighting that a person from the research group spent 1.5 months in the falcons’ breeding center in order to imprint the animals used in the study, and thus, minimize further stress. Besides, she observed the environmental conditions of the animals in order to reproduce them in the isolators. Falcons were always fed by this person (since 3 weeks of age), and got used to see her, making her presence not a stressful factor. This person travelled with the animals to our laboratories, and she also carried out the sampling and other procedures during the experiment. Isolators were adapted for the falcon’s welfare – river stones were disposed all over the surface of the isolator’s floor, and elevated bars covered with coconut tappet were installed to allow the animals to clean and file their beak and nails as well as to perch in a high position, similar to field situations. Falcons were fed twice a day with dead whole prey (1-day-old commercial chicks) supplemented with vitamins. As falcons do not generally drink water in their natural environment and obtain it from the food, water was not provided. Therefore, the dead whole preys were sprayed with water before fed to the falcons.

Falcons were brought from the falcons’ breeding center to our BSL-3 facilities, and were randomly assigned into different experimental groups, each of them in separate negative-pressurized isolators, with a maximum of 4 falcons per isolator. Falcons were acclimatized to their new environment for a period of 1 week before the start of the experiment.

**Table 1.** Clinical signs monitored for supervising eventual suffering and/or stress.

| SCORE | BEHAVIOUR SIGNS. DIGESTIVE SIGNS. NEUROLOGIC SIGNS. RESPIRATORY SIGNS |
| --- | --- |
| 0 | Active, alert. Clean cloaca. Other signs not apparent. |
| 1 | Barely active, ruffled feathers. Dirty cloacal feathers. Sneezing, slight nasal exudate, slight gasping. |
| 2 | Slight apathy, alert to noises but inactive and ruffled. Stained cloacal feathers. Incoordination but able to move. Evident gasping. |
| 3 | Prostration, almost immobile, no reaction to stimulus. Blood-stained cloacal feathers. Pedaling, incoordination, opistothonus. Severe gasping and abdominal respiration. |

**Table 2.** End point criteria of the experiment.

| SCORE^1^ | CRITERIA |
| --- | --- |
| 0-4 | Possible treatment and daily monitoring |
| 4-8 | Possible euthanasia |
| >8 | Euthanasia |

^1^ When in the evaluation appears more than one 3, all 3 turn into 4.
